# Tissue Transglutaminase (TG2)-Induced Inflammation in Initiation, Progression, and Pathogenesis of Pancreatic Cancer

**DOI:** 10.3390/cancers3010897

**Published:** 2011-02-25

**Authors:** Kapil Mehta, Amy Han

**Affiliations:** 1 Department of Experimental Therapeutics, The University of Texas M. D. Anderson Cancer Center, Houston, TX 77030, USA; E-Mail: amy.han@colorado.edu; 2 Graduate School of Biomedical Sciences, The University of Texas Health Science Center, Houston, TX 77030, USA

**Keywords:** inflammation, metastasis, chemoresistance, epithelial-to-mesenchymal transition, cancer stem cell

## Abstract

Pancreatic cancer (PC) is among the deadliest cancers, with a median survival of six months. It is generally believed that infiltrating PC arises through the progression of early grade pancreatic intraepithelial lesions (PanINs). In one model of the disease, the *K-ras* mutation is an early molecular event during progression of pancreatic cancer; it is followed by the accumulation of additional genetic abnormalities. This model has been supported by animal studies in which activated *K-ras* and *p53* mutations produced metastatic pancreatic ductal adenocarcinoma in mice. According to this model, oncogenic *K-ras* induces PanIN formation but fails to promote the invasive stage. However, when these mice are subjected to caerulein treatment, which induces a chronic pancreatitis-like state and inflammatory response, PanINs rapidly progress to invasive carcinoma. These results are consistent with epidemiologic studies showing that patients with chronic pancreatitis have a much higher risk of developing PC. In line with these observations, recent studies have revealed elevated expression of the pro-inflammatory protein tissue transglutaminase (TG2) in early PanINs, and its expression increases even more as the disease progresses. In this review we discuss the implications of increased TG2 expression in initiation, progression, and pathogenesis of pancreatic cancer.

## 1. Introduction

Pancreatic cancer (PC) is among the deadliest malignancies with the worst prognosis. Worldwide, nearly 230,000 new cases of PC are diagnosed every year, and almost the same number of patients die of the disease, underscoring the aggressive nature of this cancer [1]. Major reasons for poor survival of patients with PC are the absence of specific symptoms and lack of tests that would detect the disease early, rapid metastasis, and intrinsic resistance of cancer cells to chemo- and radiation therapies. Gemcitabine (Gemzar), the drug of choice for treating PC, shows minimal benefit in terms of patients' survival. Before we can develop new therapeutic strategies that would improve these dismal clinical outcomes, a more detailed understanding of the biology of PC progression is required. Chronic pancreatitis is considered to be the major risk factor in development of PC. Approximately 70% of chronic pancreatitis cases result from excessive alcohol consumption and are characterized by destruction of pancreatic parenchyma, infiltration of inflammatory cells, and tissue fibrosis, accompanied by impaired pancreatic exocrine and endocrine functions. Similarly, patients with hereditary pancreatitis, which arises as a result of mutation in the *trypsinogen* gene on chromosome 7, are at higher risk of developing pancreatic cancer [2]. Recent evidence suggests that endotoximia, which is due to increased gut permeability in alcoholics, may trigger inflammation in the pancreas tissue and contribute to the progression of chronic pancreatitis by activating pancreatic stellate cells (PSCs). Activation of PSCs could favor malignant transformation of pancreatic ductal cells, leading to dysplasia and cancer. Smoking is another risk factor that can contribute to the development of pancreatic cancer [3]. Cigarette smoke can induce ROS production and lead to inflammation and DNA damage due to continuous exposure to carcinogens.

The loss of tumor suppressor genes, along with mutations in proto-oncogenes, has been suggested to assist in the development of pancreatic cancer [4]. There are many mutations that have been reported in pancreatic cancer, *K-ras* being the most common [5], followed by the loss of *p53* and *DPC4/SMAD* [6]. The *K-ras* mutation probably occurs early on and leads to uncontrolled cell growth. These genetic alterations can be induced by reactive oxygen species (ROS) released by activated PSCs and result in DNA strand breaks, sister chromatid exchanges, mutations, and DNA adduct formation [7]. ROS can also change the nucleotide structure and produce mismatched pairing during transcription. The major mutation caused by ROS involves the transversion of guanine to thiamine, which has been found in *K-ras* mutations [8].

PSCs play an important role in maintaining the homeostasis of the extracellular matrix (ECM) within the pancreas by regulating its synthesis and degradation. During pancreatic injury, however, these cells are activated and secrete cytokines like transforming growth factor (TGF)-β, resulting in an autocrine loop for cell activation. Moreover, activated PSCs acquire a myofibroblast-like phenotype and synthesize and secrete excessive amounts of the ECM proteins such as collagen, laminin, and fibronectin, which comprise fibrous tissue.

In recent years, molecular mechanisms and pathways that contribute to the transformation of PSCs from the quiescent to the activated state have been areas of intense research. Several signaling pathways (e.g., JAK-STAT, MAPK, NF-κB, AP-1, *etc.*) have been implicated in PSCs activation [9]. In this context, recent observations that activated PSCs, PanINs [10] and advanced-stage pancreatic cancer [11,12] express elevated levels of the pro-inflammatory protein tissue transglutaminase (TG2), in conjunction with the earlier observations that TG2-catalyzed protein crosslinking reactions play an important role in ECM stabilization [13], are of particular importance. This review will discuss the implications of TG2 expression in inflammation and its possible significance in the initiation, progression and pathogenesis of pancreatic cancer.

TG2 is a multifunctional protein, which in addition to catalyzing calcium-dependent crosslinking of proteins, can bind and hydrolyze GTP [14]. TG2 has been implicated in a number of pathological processes such as hepatic injury, degenerative disorders of neurons, myocardial hypertrophy, celiac disease, and tissue fibrosis [15]. Many biological functions of TG2 are attributed to its ability to catalyze calcium-dependent protein crosslinking reactions. However, in recent years evidence has accumulated indicating that TG2 can also function as a cell-signaling scaffold protein and regulate protein stability, cell functions and gene regulation in a context-dependent manner [16-19]. Though predominantly a cytosolic protein, TG2 can also translocate to the nucleus, cell membrane, or mitochondria and can even be secreted outside the cell.

**Figure 1.**
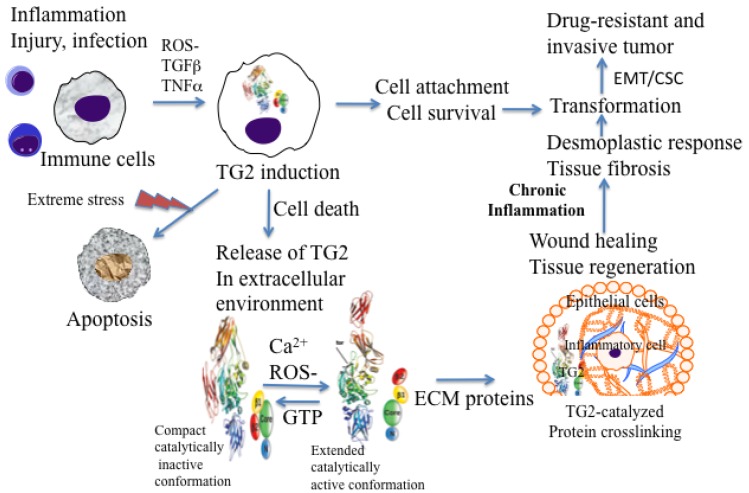
Possible role of inflammation-induced TG2 expression in initiation and progression of cancer.

Under physiological conditions, TG2 exists as a catalytically inactive intracellular protein due to low Ca^2+^ and high inhibitory concentrations of GTP/GDP [20]. In this form, TG2 acts as a scaffold protein and results in the activation of some critical cell survival signaling pathways (NF-κB, Akt, focal adhesion kinase) [16,19,21,22]. However, under extreme conditions of cellular stress or trauma, loss in Ca^2+^ homeostasis can activate intracellular TG2 resulting in crosslinking of cellular proteins and apoptosis or necrotic death of cells [23,24] (Figure 1). Thus, distinct activation of status of TG2 (compact versus extended) in a cell may be a reflection of the fact that TG2 functions as a double-edged sword in cancer cells, conferring advantage or disadvantage to the tumor.

## 2. TG2 and Inflammation

Inflammation (derived from the Latin word ‘*inflammare*’ to set on fire) is a complex biological response triggered by harmful stimuli. Inflammation involves such multiple events as cell migration, cell proliferation, synthesis and stabilization of the ECM, neovascularization, and apoptosis, all of which are needed for successful wound healing and tissue repair. Acute inflammation also referred to as therapeutic or physiologic inflammation is an initial stage of inflammation (innate immunity) mediated through activation of the immune system; it helps the body ward off infections and lasts for only a short period. If inflammation persists for a long period of time, it becomes chronic and is referred to as pathological inflammation [25]. Indeed, chronic inflammation has been linked to most chronic illnesses, including cancer, cardiovascular diseases, diabetes, obesity, pulmonary diseases and neurologic diseases [25].

Several studies have implicated TG2 in wound healing and inflammation [26–28]. Cytokines and growth factors secreted during early phases of cell injury regulate TG2 expression. For example, pro-inflammatory cytokine transforming growth factor (TGF)-β1 enhances *TG2* gene expression through a response element located 868 bases upstream of its translational initiation site [29]. TG2, in turn, binds to cell surface TGFβ1 and promotes its conversion to the biologically active form, thus creating a positive feedback loop. Similarly, TNF-α induces TG2 synthesis by activating NF-κB [30] and TG2 activates NF-κB [31], thus creating an autocrine loop. TG2 expression can also be enhanced by IL-1 [32] and IL-6 [33]. In response to cutaneous injury, TG2 expression and activity are increased at sites of neovascularization and in endothelial cells, skeletal muscle cells, and macrophages infiltrating wounds in the border between healthy and injured tissue [34].

*In vivo* studies using TG2-deficient mice revealed that these mice are better protected from lipopolysaccharide-induced septic shock than their wild-type counterparts [35]. The protective effect was associated with decreased NF-κB activation, decreased neutrophil recruitment into the kidney and peritoneum, and reduced damage to renal and myocardial tissues in TG2-deficient mice. These observations imply that TG2 expression plays a role in promoting the inflammation-induced pathogenesis of septic shock. Moreover, TG2-deficient mice have been used to determine whether TG2 plays a protective or pathologic role during liver injury. Results from these studies suggested that TG2-deficient mice were more prone to death caused by carbon tetrachloride- or alcohol-induced liver injury than the control mice indicating a protective role for TG2 in liver injury [36]. The protection of TG2 expressing mice was associated with increased inflammatory response and increased ECM accumulation. Studies to determine the involvement of TG2 in renal fibrosis also revealed that TG2-deficient mice are less susceptible to kidney damage than their normal counterparts [37]. A protective effect was associated with reduced infiltration of macrophages and myofibroblasts, decreased collagen synthesis and reduced TGF-β activation in TG2-deficient mice.

Macrophages are among the first cells that are recruited at inflammatory sites. In response to the inflammatory cytokines and other pro-inflammatory factors, macrophages accumulate large amounts of TG2 protein at these sites [38]. Generally, in intracellular sites, TG2 attains a folded or compact conformation (due to low calcium and high GTP levels) and remains catalytically inactive. However, upon its release in the extracellular environment (by passive release or due to cell death) TG2 acquires a catalytically active or extended conformation and thus catalyzes irreversible crosslinking of the ECM proteins (Figure 1). TG2-catalyzed crosslinking of ECM proteins via N′ε(γ-glutamyl)-lysine isopeptide bonds represents an important step in maturation and stabilization of such ECM proteins, such as collagen and fibronectin and is critical for the wound healing. Indeed, N′ε(γ-glutamyl)-lysine crosslinks, which are undetectable in normal liver tissue, become prominent in fibrotic livers of patients with a variety of chronic liver diseases [36]. In addition, the crosslinking ability of TG2 appears to be crucial for the activation of TGF-β, the most fibrogenic cytokine [39]. In many tissues, enhanced TG2 activity, which results from cell damage due to chronic inflammation, is required for TGF-β activation via crosslinking of large latent complexes to the cell surface, or to fibronectin and other ECM components through the latent TGF-β binding protein portion. Amine donor substrates, such as putrescine and cystamine, which act as competitive inhibitors for TG2 in its crosslinking reaction, have been shown to protect against CCl4-induced fibrosis [40]. Thus, chronic expression of TG2 may promote cell survival and permit accumulation of oncogenic mutations, resulting in cell transformation and tumor progression (Figure 1).

## 3. TG2 in Pancreatic Cancer

**Figure 2.**
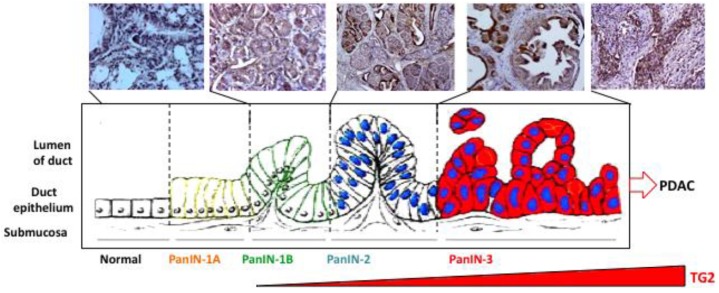
Progressive increase in TG2 expression during the course of pancreatic cancer progression in mice carrying pancreas-specific oncogenic K-*ras^K12D^*. A similar increase in TG2 expression has been observed in tumor samples from pancreatic cancer patients [10].

Pancreatic carcinoma is believed to develop from histologically identifiable intra-ductal lesions known as PanINs that follow a series of structural, cytological, and genetic premalignant changes. Cheung *et al.* [10] recently observed that 60% of preneoplastic lesions (PanIN-1, 2, and 3) in tumor samples from patients expressed elevated TG2 levels. In a preliminary study using tissue samples from transgenic mice carrying pancreas-specific oncogenic K-*ras^g12d^* mutation, we observed a similar increase in TG2 expression in early/late-stage PanINs and in invasive carcinoma (Figure 2). Similarly, gene analysis of tumor samples revealed that TG2 is among the highest expressed genes in pancreatic cancer [12]. Moreover, our own studies demonstrated that basal expression of TG2 is significantly high in PC cell lines as well as in tumor samples from PC patients than in normal ducts (*P* < 0.0001) [11]. Importantly, high TG2 expression in tumor samples is associated with nodal metastasis (*P* = 0.017) and lymphovascular invasion (*P* = 0.045) [11]. These observations suggest that aberrant expression of TG2 occurs early in pre-neoplastic lesions in the pancreas and increases as the disease progresses. Using the gain- and loss-of-function approaches, several studies have supported a role of TG2 in drug resistance and metastasis of PC cells. Thus, high-TG2-expressing Panc-28 PC cells showed minimal growth inhibitory effect (IC_50_ =10 μM) in response to gemcitabine treatment when compared with the TG2-deficient BxPC3 cells (IC_50_ = 0.1 μM). As is the case for PC cells, the development of drug resistance in breast cancer, melanoma, lung carcinoma, and ovarian carcinoma has been associated with increased TG2 expression [16]. Downregulation of TG2 by antisense or siRNA resulted in reversal of drug resistance in these cells.

Cell motility and invasion processes are intimately related to metastasis. TG2 expression has been linked to enhanced invasive potential of PC cells in a Matrigel invasion assay. High-TG2-expressing Panc-28 cells showed three-fold higher invasion than the low-TG2-expressing BxPC3 cells.

Moreover, downregulation of TG2 by siRNA dramatically compromised the ability of Panc-28 cells to invade through Matrigel transwell inserts. Conversely, the infection of low-TG2-expressing BxPC-3 cells with TG2-adenoviral construct rendered the cells four-fold more invasive than non-infected or adenovirus-alone-infected cells [11].

Downregulation of TG2 in PC cells has been associated with autophagic death (type II programmed cell death). Thus, transfection of Panc-28 or Capan-1 cells with TG2 siRNA (but not control siRNA) induced massive accumulation of autophagic vacuoles, as revealed by phase-contrast microscopy [41]. The vacuoles were highly acidic, as determined by acridine orange staining, and were accompanied by the accumulation of microtubule-associated light chain (LC3) protein, hallmarks of cells undergoing autophagy. Transmission electron microscopy (TEM) revealed the formation of autophagosomes with cellular organelles in TG2 siRNA-transfected Panc-28 cells. In addition to autophagic vesicles, TEM images also revealed the merging of autophagic vesicles with lysosomes and mitochondria, suggesting lysosomal-mediated degradation of cellular organelles. These results suggested that TG2 expression protects PC cells from autophagic death. Although the role of autophagy in cell death has been controversial, recent reports have noted that prolonged autophagy can lead to cell death. Indeed, the autophagy regulator gene, *BECN1*, is considered to be a haploinsufficient tumor suppressor gene that induces autophagy when overexpressed. From these results, it is reasonable to believe that downregulation of TG2 in PC cells blocks cell survival signaling and thus leads to autophagic death. Taken together, these results suggested a novel role for TG2 in protecting PC cells from cell death.

## 4. Epithelial-to-Mesenchymal Transition (EMT)

### 4.1. EMT in Pancreatic Cancer Cells

Cancer shares many similarities with inflammatory response and tissue injury. Rudolph Virchow provided the first indication of a possible link between inflammation and cancer. He proposed that cancer originates at the sites of chronic inflammation and that some classes of irritants together with tissue injury and inflammation may enhance cell proliferation and cancer progression [42]. This idea remained quiescent for many years until several lines of evidence, from epidemiological studies to molecular studies of genetically modified mice, led to a general acceptance that inflammation and cancer are closely linked. For example, epidemiological studies supported the contention that individuals with chronic inflammation are predisposed to various types of cancer. Thus, ulcerative colitis, chronic gastritis, hepatitis, and chronic pancreatitis and their respective associations with colon, gastric, liver, and pancreatic carcinomas represent a few examples. Moreover, anti-inflammatory drugs are known to reduce the risk of certain cancers. Thus, it is becoming clear that the proliferation of cells alone is not enough to cause cancer. Sustained cell proliferation of oncogenically transformed cells in an environment rich in inflammatory cells, growth factors, activated stroma, and DNA-damage promoting agents is needed for successful neoplastic growth and spread.

**Figure 3.**
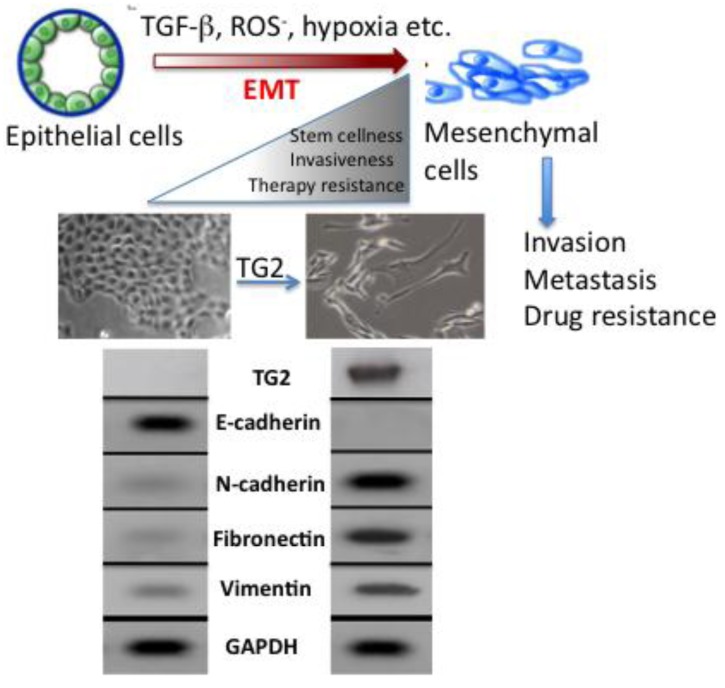
Epithelial-to-mesenchymal transition (EMT) causes functional phenotypic transition of polarized epithelial cells into migratory mesenchymal cells. Stable expression of TG2 induces EMT in epithelial cells (shown here are mammary epithelial MCF10A cells) as revealed by the loss of E-cadherin, upregulation of N-cadherin, fibronectin, and vimentin, anchorage-independent growth, increased invasion, and resistance to doxorubicin [48].

The molecular mechanisms that link inflammation and cancer have remained elusive until recently. Multiple studies of molecular pathways involved in inflammation and cancer have pointed to the EMT as a common link between inflammation, organ fibrosis, and cancer [43,44]. The EMT marks the conversion of polarized epithelial cells into highly motile fibroblastoid-like cells and involves the loss of epithelial cell-cell junctions and modification of the cytoskeleton (Figure 3). The reduction in intracellular cohesion is mainly the result of alterations in the intracellular junctions composed of desmosomes, adherens junctions, and tight junctions. Epithelial cells use E-cadherin as a major protein in adherens junctions and promote its interaction with the extracellular domain of another E-cadherin molecule from a neighboring cell. During EMT, the activity of the adherens junctions is substantially modified, predominantly owing to the replacement of E-cadherin by N-cadherin, a process called “cadherin switching”.

During embryogenesis, the EMT plays an essential role in tissue development [43]. Its reactivation in adult tissues can be a physiologic attempt to control inflammatory response and to heal damaged tissue. However, in a pathological context, such as in tumors or during organ fibrosis, this healing response may be harmful and result in metastasis and organ failure [44]. Indeed, in recent years evidence has mounted in support of the notion that EMT plays a critical role in cancer progression by promoting invasion and metastasis of localized carcinoma [45]. The cells with EMT acquire the ability to degrade the basement membrane (due to increased activity of matrix metalloproteases MMP2, MMP3, and MMP9) and migrate through the ECM to populate different areas during embryogenesis or cancer progression, or to behave like profibrotic myofibroblasts in the interstitial spaces between tissues. Certain elements, such as TGF-β1 and hypoxia that are known for their role in the control of inflammation and induction of tumor cell death, can act as inducers of the EMT through a complex network of effectors [46,47]. For example, TGF-β proteins can activate both Smad and non-Smad signals, which then can crosstalk with other signaling pathways to provide context-dependent outcomes. The context-specific effects can be generated through activation of distinct pathways for different durations and changes in the levels of interacting protein partners and availability of repressors and activators. Although our understanding of the molecular mechanism of EMT has significantly advanced during the past decade, much work needs to be done to define the transcriptional regulatory networks and the key target genes that drive EMT in a context-specific manner. In this regard, our recent findings that TGF-β-induced EMT in mammary epithelial cells is completely dependent on the expression of TG2 [48] is of great significance.

### 4.2. TG2-InducedEMT

As discussed earlier, multiple cancer cell types exhibiting resistance to chemotherapy or isolated from metastatic sites express high basal levels of TG2 [16,19]. Notably, in pancreatic cancer cells TG2 expression has been associated with constitutive activation of FAK, Akt, and NF-κB and down-regulation of the tumor suppressor protein PTEN [49,50]. TG2-mediated activation of these oncogenic pathways probably contributes to the increased invasiveness and resistance to chemotherapy in cancer cells. Indeed, downregulation of TG2 by small-interfering RNA (siRNA) compromised the ability of pancreatic cancer cells to invade and metastasize both *in vitro* and in a mouse model [11,50]. Similar effects of TG2 inhibition on drug sensitivity and metastasis of ovarian [51], lung [52], malignant melanoma [53], and glioblastoma [54] cancer cells have been observed. Understanding of TG2-reguated pathways that contribute to increased cell survival and invasiveness in PC may offer novel therapeutic targets for intervention and effective treatment of the diseases.

Acquisition of EMT is considered to be an important factor in development of drug resistance by pancreatic cancer cells [55]. Several pancreatic cancer cell lines with high expression of epithelial markers (e.g., E-cadherin) and low expression of mesenchymal markers (e.g., Zeb1) are sensitive to chemotherapeutic drugs. Conversely, the cell lines with high mesenchymal markers are resistant to these drugs [56]. Similarly, pancreatic cancer cells that are resistant to gemcitabine exhibit strong expression of EMT markers [57]. Consistent with these results is the observation that high expression of TG2 is associated with increased resistance of pancreatic cancer cells to gemcitabine while low TG2 expressing cells are sensitive to this drug [11]. Based on this information, it is tempting to speculate that TG2 expression may contribute to drug resistance by modulating the EMT. Indeed, our recent data support this contention and revealed that stable expression of TG2 in mammary epithelial cells is associated with induction of EMT. Stable expression of TG2 resulted in loss of epithelial markers (E-cadherin) and gain of mesenchymal markers (vimentin, fibronectin, N-cadherin, *etc.*). Moreover, TG2 expression increased the invasiveness of MCF10A cells through the Matrigel matrix and resulted in the loss of apical-basal polarity of cells as determined in 3D cultures [48] (Figure 3). In addition, TG2 expression resulted in increased cell survival and anchorage-independent growth of mammary epithelial cells and induced the expression of transcriptional repressors *Snail1*, *Zeb1*, *Zeb2* and *Twist1* [48]. Shao *et al.* [58] reported similar observations in ovarian cancer cells where TG2 expression was shown to induce the EMT and this modulation contributed to increased invasiveness and metastasis of ovarian cancer cells. Using the loss- and gain-of-function approach, these authors concluded that TG2 induces a mesenchymal phenotype (characterized by a cadherin switch) to promote invasive behavior in ovarian cancer cells. This transition was associated with altered expression of the transcriptional repressor *Zeb1*. Recently, it has been proposed that EMT can enable cancer cells not only to survive in stressful environments but also to acquire the ability of self-renewal by inducing a stem cell state [59]. Cancer stem cells have the ability to self-renew, differentiate, and thus to drive the expansion of malignant cells with invasive and metastatic properties [55]. Moreover, this small subpopulation of cancer stem cells exhibit high resistance to drugs, which might explain why cancer is difficult to eradicate completely. Indeed, drug-resistant cells from patients with pancreatic cancer are highly tumorigenic and have greater metastatic potential than cells that are drug sensitive [59,60]. Based on these observations, it has been proposed that current therapeutic strategies kill only differentiated cells, while sparing the cancer stem cell population. Thus, the concept of cancer stem cells has offered new insights in the process of tumor progression and might offer novel targets for prevention of cancer recurrence and possibly cancer treatment. Cancer stem cells have been identified and isolated from a number of solid tumors, including breast, lung, pancreas, prostate, colorectal and brain [61]. Our initial data suggest that TG2-induced EMT in mammary epithelial cells could confer stem cell phenotype as determined by enrichment of CD44^high^/CD24^low/-^ subpopulation [48]. Further studies are needed to characterize the complex molecular network through which TG2 modulates the EMT and stem cell characteristics in epithelial cells.

As previously discussed, aberrant expression of TG2 in epithelial cells results in constitutive activation of FAK, Akt, and NF-κB [21,22,31,49]. These pathways are known to be intimately involved in the regulation of EMT, conferring drug resistance, and promoting metastasis. For example, activated NF-κB is considered to be a hallmark of many advanced-stage tumors [62]. Thus, constitutively active NF-κB is known to confer resistance to death-inducing stimuli, including chemotherapeutic agents [63], and to promote metastasis by inducing EMT [64]. NF-κB-induction of EMT has been attributed to the increased stability of Snail owing to increased synthesis of ICOP9 signolosome 2, which blocks the ubiquitination and subsequent degradation of Snail [65]. In another report, constitutive activation of NF-κB in MCF10A cells was found to induce the EMT as a result of increased expression of *Zeb1* and *Zeb2* [66]. Based on these observations it is reasonable to believe that TG2-induced EMT may result from constitutive activation of NF-κB and subsequent increase in *Snail*, *Zeb1*, and *Zeb2* as observed in ovarian and mammary epithelial cells [48,58]. Indeed, TG2 was recently shown to associate with NF-κB for its recruitment to the promoter sequence of *Snail*, leading to its transcriptional regulation [67]. Similarly TGF-β, which is considered to be the most important EMT-inducing factor in a diverse range of tumor cells [47], can crosstalk with TG2. Thus, TGF-β induces TG2 expression [29] and TG2 can activate TGF-β [39]. Indeed, TGF-β failed to induce EMT in mammary epithelial cells that were rendered TG2-deficient by stable transfection with shRNA prior to TGF-β treatment [48]. These results clearly imply that TG2 is an important downstream mediator of TGF-β -induced EMT. It is conceivable that TG2 represents a converging point for TGF-β induced non-canonical signaling that is considered critical in induction of the EMT and supports TG2 as a common link between inflammation and cancer.

Based on these observations we propose a model in which continuous production of mediators such as, ROS-, TGF-β, and IL-6 at smoldering inflammatory sites (due to chronic infection, tissue injury, or tumor growth) aberrantly upregulate TG2 expression, which then orchestrates multiple downstream signals to affect such critical processes as EMT and acquisition of stem cell like (CSC) characteristics in epithelial cells. Altered homotypic (cell-cell) and heterotypic (cell-ECM) interactions that follow the loss- and gain- of EMT/CSC-related genes confer cell growth and cell survival advantages, increase the synthesis of ECM proteins and increase the ability of cells to migrate and invade the surrounding tissue. These changes result in a fibrotic response, desmoplastic response, or transformation of primary tumor into metastatic tumor. Thus, aberrant expression of TG2 in cancer cells represents a promising therapeutic target for reversing drug resistance and inhibiting the metastatic spread of PC.

## 5. TG2 as a Therapeutic Target

Based on the information discussed in preceding sections, we concluded that inhibition of TG2 could be a promising therapeutic target for reversing intrinsic resistance of pancreatic cancer cells to chemotherapeutic drugs. Using DOPC liposomes as the delivery system, we showed that TG2 siRNA could effectively downregulate TG2 expression in orthotopically growing pancreatic tumors in a nude mouse model and inhibit their growth and metastatic spread [50]. Downregulation of TG2 rendered pancreatic tumors sensitive to gemcitabine treatment.

To determine potential mechanisms underlying the antitumor effect of TG2 siRNA-DOPC, we examined its effect on several biological end-points, including cell proliferation (Ki-67), angiogenesis (CD31), and apoptosis (TUNEL). More than 90% reduction in Ki-67 expression was evident (*P* < 0.001) in tumors obtained from mice given TG2 siRNA-DOPC alone or in combination with gemcitabine. Tumors obtained from mice given gemcitabine alone or control siRNA-DOPC consistently showed high levels of Ki-67 staining [50]. We also evaluated the blood vessel density in tumors obtained from mice treated with control siRNA-DOPC, gemcitabine, TG2- siRNA-DOPC or a combination of gemcitabine plus TG2-siRNA-DOPC. We found a significant decrease in the mean blood vessel density in tumors recovered from mice that received TG2 siRNA-DOPC or gemcitabine alone. Densitometric analysis showed a significant difference in the mean microvascular density (*P* < 0.0013) in the mice that received TG2 siRNA-DOPC plus gemcitabine. Finally, we evaluated apoptosis in orthotopic tumors using TUNEL assay. Pancreatic tumors treated with gemcitabine alone or in combination with TG2 siRNA-DOPC showed significant increases in the numbers of apoptotic cells. No differences in the apoptotic index in TG2 siRNA-DOPC-treated tumors were evident, and the extent of apoptosis in this group was similar to that in the control siRNA-DOPC group.

In conclusion, our studies have provided the proof-of-concept that therapeutic delivery of TG2-specific siRNA to pancreatic tumors can be achieved using nanoliposomes. Two recent reports have documented a similar effect of liposomal delivery of siRNA to silence the expression of the oncoprotein EphA2 [68] and FAK [69]. Using this approach, both studies documented encouraging responses to the treatment of ovarian tumors in mice. Thus, the significance of elevated TG2 expression and its role in promoting the drug resistance and metastatic phenotype can be rapidly translated into the clinical setting for treatment of aggressive forms of cancer, including pancreatic cancer.
